# Targeted Delivery of Erythropoietin by Transcranial Focused Ultrasound for Neuroprotection against Ischemia/Reperfusion-Induced Neuronal Injury: A Long-Term and Short-Term Study

**DOI:** 10.1371/journal.pone.0090107

**Published:** 2014-02-28

**Authors:** Sheng-Kai Wu, Ming-Tao Yang, Kai-Hsiang Kang, Houng-Chi Liou, Dai-Hua Lu, Wen-Mei Fu, Win-Li Lin

**Affiliations:** 1 Institute of Biomedical Engineering, College of Medicine and College of Engineering, National Taiwan University, Taipei, Taiwan; 2 Institute of Pharmacology, College of Medicine, National Taiwan University, Taipei, Taiwan; 3 Division of Medical Engineering Research, National Health Research Institutes, Miaoli, Taiwan; 4 Department of Pediatrics, Far Eastern Memorial Hospital, New Taipei City, Taiwan; School of Pharmacy, Texas Tech University HSC, United States of America

## Abstract

Erythropoietin (EPO) is a neuroprotective agent against cerebral ischemia/reperfusion (I/R)-induced brain injury. However, its crossing of blood-brain barrier is limited. Focused ultrasound (FUS) sonication with microbubbles (MBs) can effectively open blood-brain barrier to boost the vascular permeability. In this study, we investigated the effects of MBs/FUS on extending the therapeutic time window of EPO and its neuroprotective effects in both acute and chronic phases. Male Wistar rats were firstly subjected to two common carotid arteries and right middle cerebral artery occlusion (three vessels occlusion, 3VO) for 50 min, and then the rats were treated with hEPO (human recombinant EPO, 5000 IU/kg) with or without MBs/FUS at 5 h after occlusion/reperfusion. Acute phase investigation (I/R, I/R+MBs/FUS, I/R+hEPO, and I/R+hEPO+MBs/FUS) was performed 24 h after I/R; chronic tests including cylinder test and gait analysis were performed one month after I/R. The experimental results showed that MBs/FUS significantly increased the cerebral content of EPO by bettering vascular permeability. In acute phase, both significant improvement of neurological score and reduction of infarct volume were found in the I/R+hEPO+MBs/FUS group, as compared with I/R and I/R+hEPO groups. In chronic phase, long-term behavioral recovery and neuronal loss in brain cortex after I/R injury was significantly improved in the I/R+hEPO+MBs/FUS group. This study indicates that hEPO administration with MBs/FUS sonication even at 5 h after occlusion/reperfusion can produce a significant neuroprotection.

## Introduction

The American Stroke Association [1] estimates that stroke accounts for 1 out of every 18 deaths and occurs every 40 seconds in the United States. In the 2012 update, the majority of strokes were ischemic (87%); 10% were intracerebral hemorrhage; 3% were due to subarachnoid hemorrhage. Ischemic stroke results from the occlusion of a cerebral artery, leading to a blocked cerebral blood flow to certain part of the brain. Two emergent clinical therapies for acute ischemic stroke are: reperfusion of the blood flow and neuroprotection of the injured brain cells. Early reperfusion within 3 h is beneficial to improve the outcome of acute human ischemic stroke. However, late recovery of circulation might cause reperfusion injury, resulting in blood-brain barrier (BBB) breakdown, or brain edema [2].

Although many animal stroke models have been developed, no single model can fully mimic clinical human stroke because of its heterogeneity. The transient three vessels occlusion (3VO, two common carotid arteries and middle cerebral artery) method provides a model for the study of ischemia-reperfusion injury [3]. This method can build a stable focal infarction in the brain. In addition, reperfusion is performed easily by untying the suture without plasminogen activator (t-PA) injection, and the effect of neuroprotection can be directly reflected in this animal model.

It has been recently reported that focused ultrasound (FUS) with microbubbles (MBs), which are ultrasound contrast agents in clinical use, can disrupt the local BBB for providing trans-vascular delivery of macromolecules [4], [5]. The mechanism of MBs/FUS-induced vascular permeability change may be caused by the opening of tight junction [6]. This disruption of BBB is transient and reversible within several hours [5], [7]. In recent study, MBs/FUS has been used to facilitate the delivery of liposomal doxorubicin into normal animal brains by opening the BBB[8]. The benefits of this delivery method have been demonstrated in animal models with brain tumors [9]–[11] and Alzheimer's disease [12], [13]. Although MBs/FUS may damage the brain parenchyma, a safe sonication can be achieved by regulating ultrasound sonication and the dosage of MBs [4], [14].

Erythropoietin (EPO) is a secreted glycoprotein produced primarily by the kidney and is used clinically to treat anemia [15], [16]. EPO is induced by hypoxia within the central nervous system. It has been reported that EPO is a promising acute therapeutic agent for cerebral ischemia in animal studies [17]. The protective mechanisms may include the activation of endogenous survival pathways that inhibit apoptosis and further reduce inflammatory responses [18], [19]. Systemic administration of EPO after induction of focal cerebral ischemia has been demonstrated to exert a potential neuroprotective effect on the outcome of stroke; however, there is a limited therapeutic time window. The best application time is up to 3 h after ischemia with a leaky BBB [20].

The aim of this study is to investigate the feasibility of utilizing FUS with MBs to deliver hEPO to ischemia/reperfusion injured rat brains beyond the conventional therapeutic time window and to examine the efficacy of this treatment in both acute and chronic phases.

## Materials and Methods

All the experimental protocols were approved by Institutional Animal Care and Use Committees of Medical College, National Taiwan University.

### Three Vessels Occlusion (3VO) Model

Male Wistar rats (230 to 250 g) were used in this study. The available data suggest that the 3VO model provides more consistent cortical injury compared to the MCAO model [21]. In this study, we employed the 3VO model to form a focal cortical infarction, and this kind of infarction is more suitable for the evaluation of the BBB opening with microbubbles/focused ultrasound (MBs/FUS). Temporary focal ischemia were based on the model described by Chen et al [22]. The rats were anesthetized by exposure to 1 to 3% isoflurane, and two common carotid arteries (CCAs) were occluded by artery clips. A burr hole was drilled at the anterior junction of the zygoma and the squamosal bone, and the exposed middle cerebral artery (MCA) was tied with a 10-0 suture. The above procedures were conducted within 10 to 15 minutes. Rectal temperature was maintained at 37±0.5°C. After an occlusion of 50 min, the suture was untied and the reflow of the right MCA and two CCAs was confirmed under a microscope.

### Experimental Grouping

**Figure 1:**
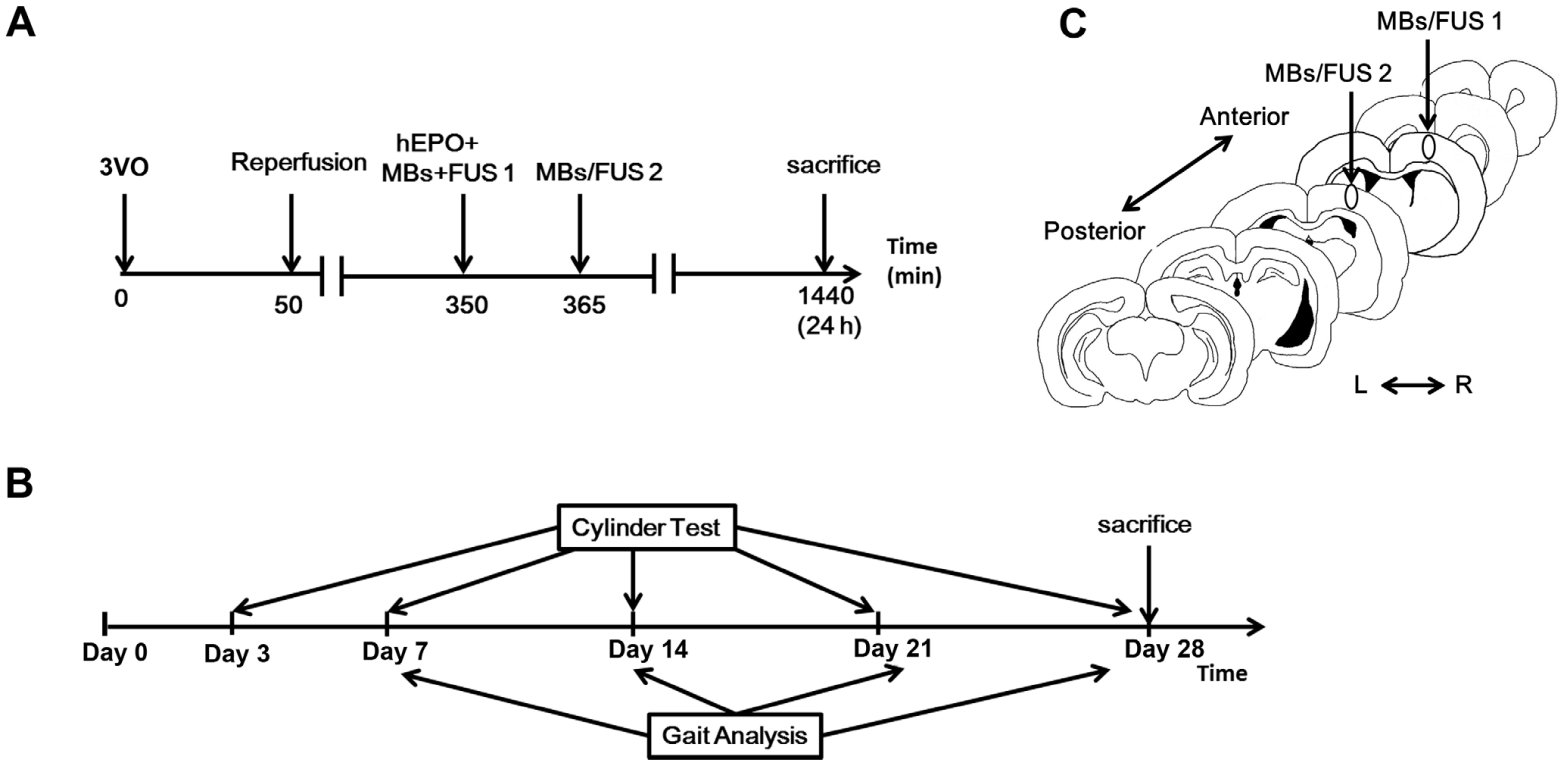
Experimental procedures. (A) For the hEPO+MBs/FUS group in the acute phase study, hEPO (i.v. injection) and first MBs/FUS were applied approximately 5 h after a 50-min 3VO. A second MBs/FUS was given 15 min later. Animals were sacrificed 24 h after 3VO. (B) In the chronic study, experimental groups were tested for motor deficits with gait analysis and cylinder test at indicated time points. Animals were sacrificed on Day 28. (C) Coronal brain section showed the cortex locations with a 2-mm interval for the two MBs/FUS application.

The experiments in this study include three parts: hEPO quantification in brain tissues, acute response and chronic response following ischemia/reperfusion (I/R). First, to quantify the amount of hEPO entering the sonicated brain, normal rats were divided into two groups: received hEPO (CBC #329871, Merck KGaA, Darmstadt, Germany) (5000 IU/kg, i.v. injection) only or hEPO plus MBs/FUS. The procedure was shown in Fig. 1A. Second, to examine the neuroprotective effect of the execution of hEPO and MBs/FUS on I/R, rats were randomly divided into four groups. Group A (denoted as I/R (Control)): rats received a 50-min 3VO. Group B (I/R+MBs/FUS): a 50-min 3VO, and then received twice MBs/FUS at 5 h after reperfusion. Group C (I/R+hEPO): a 50-min 3VO, and then received hEPO alone at 5 h after reperfusion. Group D (I/R+hEPO+MBs/FUS): a 50-min 3VO, and then received hEPO plus MBs/FUS at 5 h after reperfusion. The flowchart was displayed in Fig. 1B. Third, to evaluate the chronic response, rats were randomly divided into four groups: Group A (denoted as sham control), Group B (I/R), Group C (I/R+hEPO), and Group D (I/R+hEPO+MBs/FUS). The investigation of long-term response included: cylinder test and automated gait analysis. The time courses were shown in Fig. 1C.

### Focused Ultrasound (FUS) Sonication

A 480 KHz FUS transducer with a diameter of 10 cm, 10 cm radius of curvature was used. The acoustic beam was transmitted to the brain directly by a removable cone replete with degassed water. The FUS was precisely targeted using a stereotaxic apparatus (Stoelting, Wood Dale, IL) and the center of the focal spot was about 1 mm below the cone tip. The FUS transducer was driven by a power amplifier (500-012, Advanced Surgical Systems, Tucson, AZ) connected to a function generator (33220A, Agilent, Palo Alto, CA). The rats were laid prone beneath the cone tip, and ultrasound transmission gel (Pharmaceutical Innovations, Newark, NJ) was used to maximize the transmission of ultrasound to the brain. The focal zone is 3 mm and 13 mm in diameter and length, respectively. Pulsed sonication was applied with a peak negative pressure of 0.57 MPa, a burst length of 10 ms, a duty cycle of 1%, and a repetition frequency of 1 Hz. The duration of each sonication was 20 s. MBs (microbubbles) (SonoVue, Bracco, Amsterdam, The Netherlands) was injected as a bolus (10 µl/kg) about 15 s before each sonication. The FUS was delivered at two locations with 2 mm apart in the right hemisphere cortex: 4 mm lateral to the bregma and 1 mm or 3 mm posterior to the bregma, respectively, and both 1 mm below the skull surface (Fig. 1C).

### Quantification of hEPO Entering the Brain Tissue

CSF sample was obtained at 3 h after the execution of hEPO+MBs/FUS or hEPO alone. The rats were then perfused with saline and decapitated, and the brain was removed and sliced into six coronal sections. The sonicated region of each section was dissected and the quantity of hEPO in the sonicated brain tissue was measured by ELISA method using Quantikine human erythropoietin kit (R&D Systems, Minneapolis, MN), which did not cross-react with rat EPO

### Infarct Volume and Residual Brain Volume Evaluation

The infarct volume was analyzed 24 h after ischemia. Six consecutive coronal sections with 2 mm thick each were sliced from the frontal tip with the aid of a rat brain matrix and immersed in a 2% solution of 2,3,5-triphenyltetrazolium chloride (TTC). The stained brain sections were then fixed by immersion in phosphate-buffer containing 4% paraformaldehyde. Section images were analyzed with ImageJ (National Institutes of Health, Bethesda, MD) to calculate the infarct volume. The residual brain volume was analyzed one month after I/R. The brains were removed and sliced into six consecutive coronal sections with 2 mm thick. Section images were analyzed with ImageJ to calculate the residual brain volume.

### Behavioral Evaluation

The neurological status of the rats was evaluated 24 h after ischemia. Neurological score was based on Menzies behavioral function [23]. Score from 0 to 4 represents the extent of damage from normality to severity. Score 0: rats can extend both forelimbs; score 1: the contralateral forelimb is consistently flexed during suspension; score 2: decreased grip of the contralateral forelimb when pulled by the tail; score 3: rats show a mono-directional circling at a slight jerk of the tail; and score 4: a consistent circling occurs. One author (H.-C. Liou) blind to the treatment condition performed the neurological evaluation. To quantify the asymmetric forelimb use for the stroke animals, the cylinder test was performed on Day-3, Day-7, Day-14, Day-21, and Day-28 after I/R. The animals were placed in a 20-cm-diameter cylinder with transparent glass and at least 25 contacts of the forelimbs on the wall of the cylinder were recorded for each rat[24]. The contact percentage of impaired forelimb was expressed as contacts of impaired forelimb divided by total contacts. The theoretical value was 50% for the sham group. Animals were subjected to gait measurement every week for one month after I/R utilizing CatWalk-automated gait analysis system (Noldus Information Technology, Wageningen, The Netherlands)[25], [26]. For gait assessment, the animals were subjected to three consecutive runs. After identifying each footprint, the images were converted into digital signals and stroke-related gait data were generated including intensity of paws (pressure) and angle of the paw axis relative to the body axis (inward angles expressed as minus; outward angles as plus).

### Immunohistochemistry

Immunohistochemical staining was obtained both 24 h and 28 days after 3VO. The rats were perfused with saline and then fixed with phosphate buffer containing 4% paraformaldehyde. The brain was removed, post-fixed with 4% paraformaldehyde at 4°C overnight, and then stored in a 30% sucrose solution at 4°C for two days. Brain tissue slices (30 µm thick) were pretreated with 3% hydrogen peroxide to block endogenous peroxidase activity before incubation of primary antibody. After blocking in 4% non-fat milk containing 1% Triton X-100 for 1 h, brain tissue slices were incubated overnight at 4°C with the following primary antibody: NeuN (1∶500; Millipore, Billerica, MA), CD-11b (1∶500; AbD Serotec, Oxford, UK), and GFAP (1∶500; Millipore, Billerica, MA) in PBS. After a brief wash, brain tissue slices were then incubated with horse anti-mouse biotinylated secondary antibodies, and processed with avidin-biotin complex system (ABC kit; Vector Laboratories, Burlingame, CA), which was visualized by incubating with 0.5% diaminobenzidine and 0.01% hydrogen peroxide in PBS. Finally, the brain tissue slices were washed in PBS and mounted on slides. Usually microglia activation reaches the peak at ∼72 h after ischemia, and some reports demonstrated that microglia activation may appear as early as 24 h after ischemia [27], [28]. In this study, we like to display the delivery of hEPO into the sonicated brain tissue and to see its resulting effect as early as possible, and hence we performed the difference between I/R and I/R+hEPO+MBs/FUS groups at 24 hr after ischemia. For detecting Nissl body in the cytoplasm of neurons, the brain was fixed with 4% paraformaldehyde, embedded in paraffin, and then was sectioned. The brain sections were sequentially conducted with the following steps: deparaffinized in xylene for 10 min, hydrated in 100% ethanol for 10 min, in 95% ethanol for 5 min, in 70% ethanol for 5 min, rinsed in water for 2 min, stained in a 0.1% cresyl violet solution for 20 min, and then rinsed in water. After dehydration with ethanol, sections were mounted with xylene-based mounting solution.

### Statistical Analysis

Values are expressed as mean ± S.E.M. The results were analyzed with one-way ANOVA with post hoc test. Statistical significance was defined as p<0.05.

## Results

### Amount of hEPO Delivered into Brain and CSF by MBs/FUS

**Figure 2:**
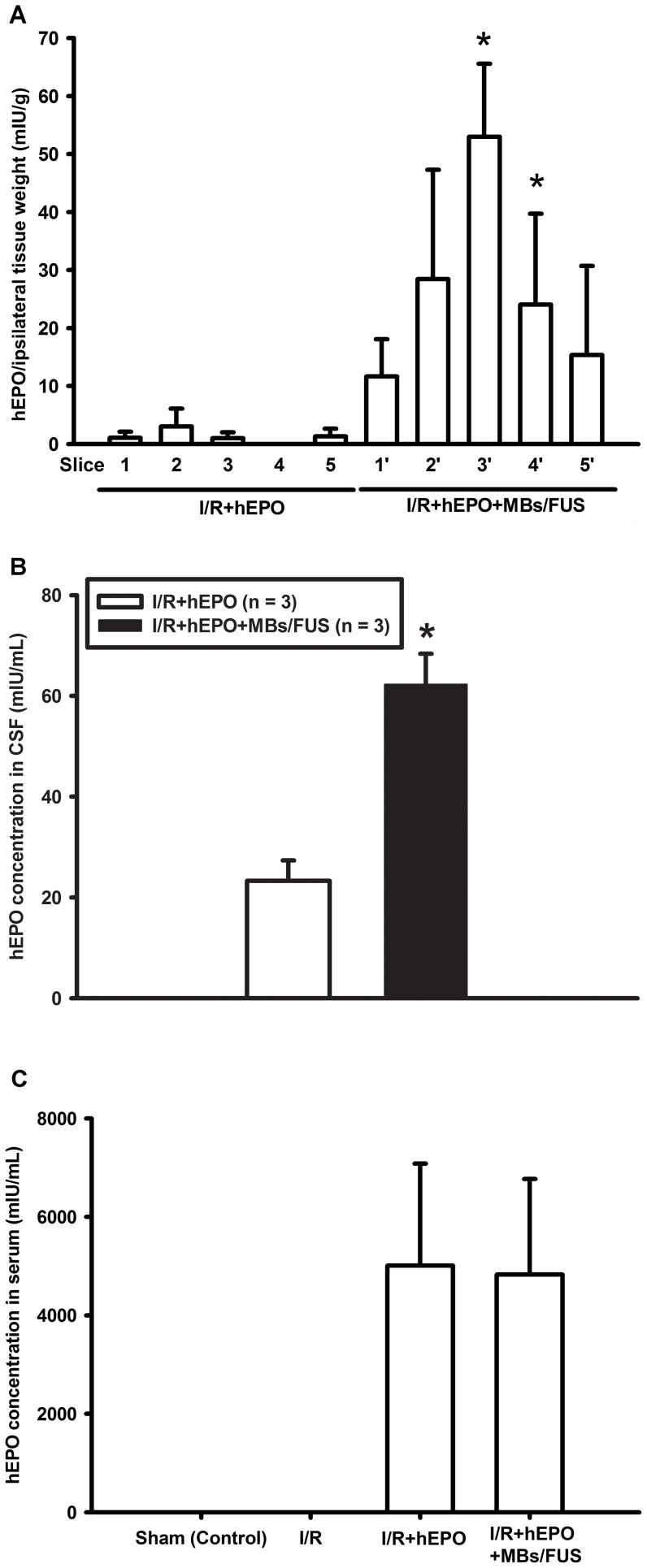
Enhancement of hEPO delivery into the brain tissues by MBs/FUS. hEPO and MBs were intravenously administered and FUS was transcranially applied. (A) Rat brains were perfused and sliced into sections at 3 h after hEPO injection. Sonicated region of the brain was dissected and quantified. The hEPO levels in sections 3 and 4 were significantly higher in the I/R+hEPO+MBs/FUS group compared to the I/R+hEPO group (n = 3 for each). Data were given as means ± S.E.M., * p<0.05, as compared with the I/R+hEPO group. (B) CSF was sampled at 3 h after hEPO injection. The hEPO concentration of CSF in the I/R+hEPO+MBs/FUS group showed significant enhancement compared with the I/R+hEPO group. (C) The serum hEPO was also sampled at 3 h after hEPO injection. No hEPO was found in the sham and I/R groups without hEPO injection.

To study the quantity of hEPO delivered into brain, hEPO was intravenously injected first and then MBs/FUS were applied twice at a 15 min interval on the right cortex (Fig. 1D). The hEPO levels in the brain sections were measured at 3 h after hEPO injection. It was found that hEPO levels in sections 3 and 4 of the cortex were significantly higher in the hEPO+MBs/FUS group (n = 3) than in the hEPO group (n = 3) (Fig. 2A). These results indicate that sonication with microbubbles increased the entry of hEPO through the BBB. The hEPO concentration of CSF in the I/R+hEPO+MBs/FUS group showed a significant enhancement compared with the I/R+hEPO group (Fig. 2B). The serum hEPO was also sampled at 3 h after hEPO injection, and the results showed that both groups had quite high levels of hEPO. No hEPO was found in both the sham and I/R groups indicated that hEPO ELISA kit did not cross-react with rat EPO.

### Reduction of Infarct Volume by hEPO+MBs/FUS

**Figure 3:**
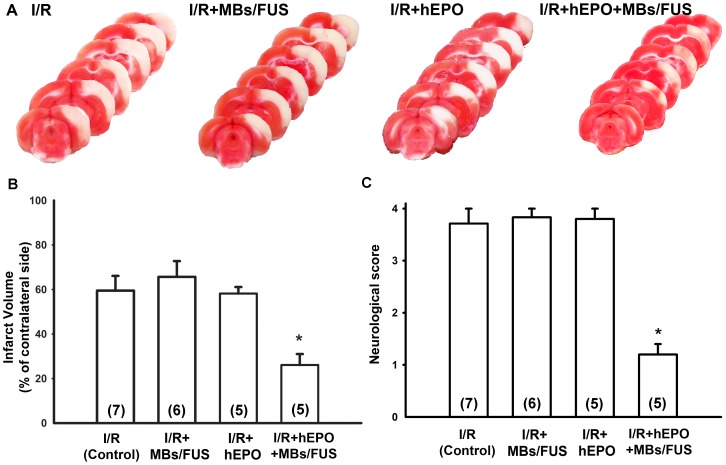
Reduction of brain infarct volume and neurological score by hEPO+MBs/FUS in rat experiments. (A) All rats were induced ischemia by 3VO for 50 min. The brain was removed and stained with TTC 24 h after 3VO. Representative sections of TTC-stained coronal brain showed the infarct area 24 h after 3VO for the I/R (control), I/R+MBs/FUS, I/R+hEPO, and I/R+hEPO+MBs/FUS groups. (B) Infarct volume was presented as the percentage of contralateral cortex. Note that the infarct volume was significantly reduced in the hEPO+MBs/FUS group. (C) Neurological score was performed 24 h after I/R. Note that hEPO combined with MBs/FUS significantly reduced the neurological scores. Data were presented as mean ± SEM, * p<0.05 as compared with the control group.

Rats were induced cerebral infarct by 3VO for 50 min, followed by reperfusion. The infarction was demonstrated by TTC staining with white color in infarct area and red color in non-infarct area (Fig. 3A). The ratio of infarct volume was presented as percentage of contralateral side of cortex. The infarct region was 59.5±6.62% (n = 7), 66.16±7.05% (n = 6), 58.62±2.93% (n = 5), and 26.87±4.92% (n = 5) in the I/R, I/R+MBs/FUS, I/R+hEPO, and I/R+hEPO+MBs/FUS groups, respectively. The I/R+hEPO+MBs/FUS group displayed a significant reduction of infarct volume, as compared with the I/R and I/R+hEPO groups (p<0.05, Fig. 3B). No significant difference was observed among the I/R, I/R+MBs/FUS, and I/R+hEPO groups. These results indicate that the enhancement of hEPO entry into ischemic area by MBs/FUS exerted neuroprotection against I/R-induced neuronal injury.

### Improvement of Neurological Behavior

The neurological scores were evaluated at 24 h after brain I/R. It was found that treatment with hEPO+MBs/FUS significantly improved neurological function (p<0.05, Fig. 3C), while treatment with hEPO or MBs/FUS individually did not show any significant difference as compared with the I/R group.

### Neuroprotective Effect of hEPO+MBs/FUS

**Figure 4:**
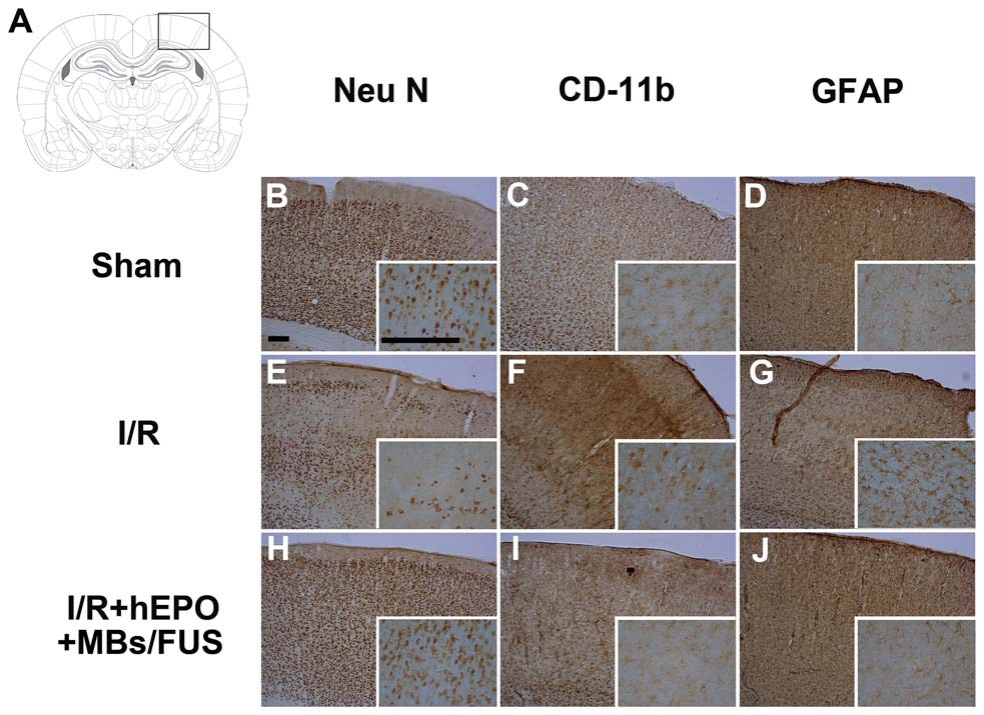
hEPO+MBs/FUS inhibits the ischemia/reperfusion-induced neuronal death and inflammation in rat experiments. Immunohistochemical staining of NeuN, CD-11b, and GFAP was performed 24 h after I/R. (A) illustrated the position of FUS sonication. In NeuN staining (B, E, and H), the I/R group showed a marked loss of neurons, whereas, neurons were intact in the sham and I/R+hEPO+MBs/FUS groups. In CD-11b staining (C, F, and I), the I/R group showed microglia activation (condensed nuclei), whereas the sham and I/R+hEPO+MBs/FUS groups showed ramified microglia. In GFAP staining (D, G, and J), there was an increase of GFAP in the I/R group but not in the sham and I/R+hEPO+MBs/FUS groups. (Scale bar  =  200 µm).

All the brain slice samples for immunohistochemical staining were obtained 24 h after 3VO and the representative slices were shown in Fig. 4. The neuronal nuclear (NeuN) staining was used to recognize neuronal nuclei in the brain. Fig. 4E showed the marked reduction of neuronal nuclei in the I/R group. On the contrary, the sham and the I/R+hEPO+MBs/FUS group displayed an intact presence of neuronal nuclei (Fig. 4, B and H). It has been reported that microglia activation occurs following neuronal death. Neuroinflammation was thus evaluated by immunohistochemical staining for CD-11b. CD-11b positive cells with large cell bodies were observed throughout the I/R group (Fig. 4F). In contrast, the I/R+hEPO+MBs/FUS group showed more homogenous distribution of cells with long fine processes extending from small cell bodies (Fig. 4I). Glial fibrillary acidic protein (GFAP) recognized astrocytes which were also activated in stroke. Fig. 3G showed that the expression of GFAP was increased in the I/R group, whereas the astroglia activation was less in the I/R+hEPO+MBs/FUS group (Fig. 4J).

### Increase of Residual Brain Volume by hEPO+MBs/FUS in Chronic Phase

**Figure 5:**
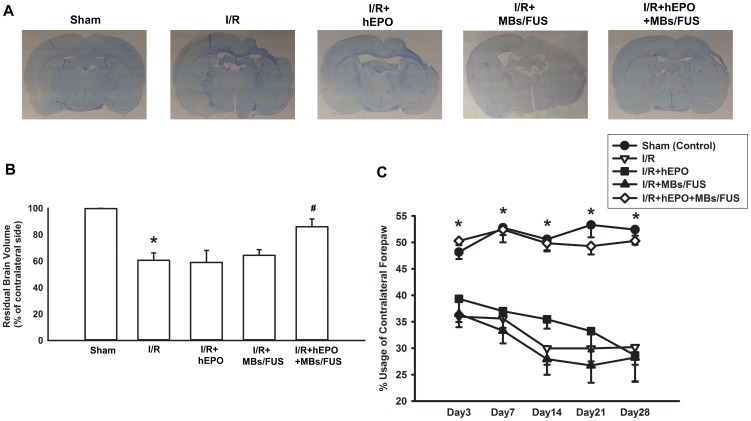
Increase of residual brain volume and improvement of limb-use in chronic phase of 3VO by hEPO+MBs/FUS. (A) Representative Nissl staining showed the residual neurons 28 days after I/R. (Scale bar  =  5 mm) (B) Residual brain volume was presented as the percentage of contralateral cortex. 28 days after I/R, residual brain volume was reduced markedly, while it was antagonized by I/R+hEPO+MBs/FUS. (C) The cylinder test was used to evaluate the rat behavior after brain injury. The I/R group showed a deficit in the usage of the left forepaw from Day-3 to Day-28, while it was reversed by hEPO+MBs/FUS. The average percentage of paw usage in normal rats was 50%. Data were presented as mean ± SEM (n = 5 for each group), * p<0.05 as compared with sham control group, # p<0.05 as compared with the I/R group.

To further study the effect of hEPO+MBs/FUS against the I/R-induced brain injury, we examined whether this treatment exerted a long-term protection. After I/R operation, the cortex volume of rat brain may gradually shrink as time goes by. Representative Nissl staining showed a drastic loss of cortex tissue in the I/R, I/R+hEPO, and I/R+MBs/FUS groups, whereas the group treated with hEPO+MBs/FUS displayed a rather intact cortex (Fig. 5A). The residual brain volume was presented as the percentage of contralateral side of cortex, and the value was 99.67±0.18% (n = 5), 60.62±5.53% (n = 5), 59.01±9.03% (n = 5), 64.41±4.29% (n = 5) and 85.97±5.85% (n = 5) for the sham, I/R, I/R+hEPO, I/R+MBs/FUS, and I/R+hEPO+MBs/FUS groups, respectively. The I/R+hEPO+MBs/FUS group displayed a significant increase of residual brain volume as compared with the I/R, I/R+hEPO, and I/R+MBs/FUS groups (p<0.05, Fig. 5B).

### Improvement of Asymmetric Limb-Use and Recovery of Gait Deficits by hEPO+MBs/FUS in Chronic Phase

**Figure 6:**
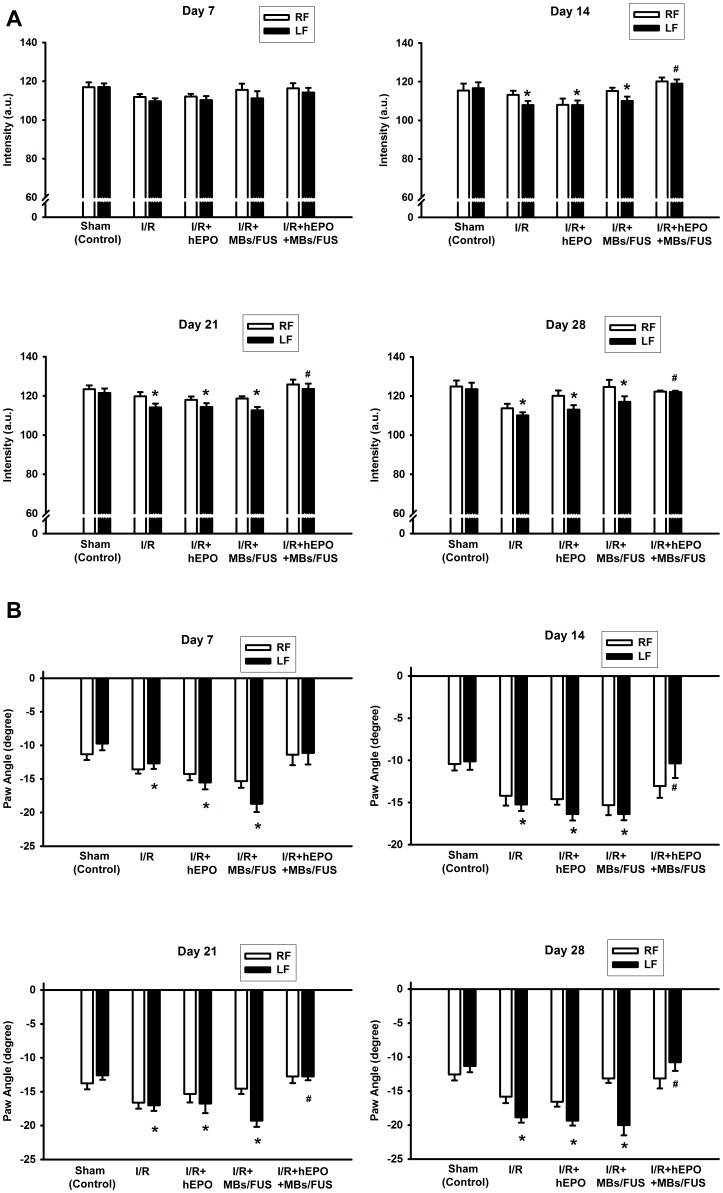
Improvement in catwalk automated gait analysis test by hEPO plus MBs/FUS in chronic phase of 3VO. Two gait analysis parameters, paw intensity (A) and paw angle (B), were assessed from Day-7 to Day-28 after I/R. In both paw intensity and paw angle, treatment with hEPO+MBs/FUS significantly improved the performance of impaired limb. Data were shown as mean ± SEM (n = 5 for each group), * p<0.05 as compared with the sham (control group), # p<0.05 as compared with the I/R group.

One month after 3VO, the behavioral tests were performed to examine the deficit of limb and there were no animals dead due to the 3VO surgery. A general linear model (GLM) with repeated measure procedure and Greenhouse-Geisser correction was used and the results showed that usage of the contralateral forepaw differed significantly among the treatment groups (F(3, 16)  = 23.602, p<0.001). Post hoc tests using the Tukey's HSD precedure revealed that the usage of the contralateral forepaws in the IR+hEPO+MBs/FUS group was significantly reduced when compared with the I/R group (Fig. 5C, p<0.05). The differences between the I/R+hEPO+MBs/FUS group and the I/R group during all the examining days were statistically significant (Dunnet's test, p<0.05 for all). Dynamic gait information was also assessed via an automated gait analysis system. In the paw-intensity measurement, the intensity of the left forepaw in the I/R group was significantly decreased from Day-7 to Day-28 as compared with the sham group (Fig. 6A), while in the I/R+hEPO+MBs/FUS group, the paw intensity significantly recovered from Day-14 to Day-28 (p<0.05). The measurement of the left-paw angle indicated that the left-paw axis was more inward in the I/R group than in the sham group (p<0.05, Fig. 6B). Treatment with hEPO+MBs/FUS had a significant recovery in the long-term response.

## Discussion

Drug treatment for brain diseases is usually hampered by the BBB, which prevents the therapeutic agents from entering the target brain tissues. Cerebral ischemia can induce BBB disruption and permit macromolecular drug to transport into the infarcted brain tissues. However, the therapeutic time window is short, and beyond this window, the efficacy of treatment is limited due to inability to achieve a sufficiently high dose of drug in the infarcted region[29]. In this study, we employed MBs/FUS to transiently open the BBB to extend the hEPO treatment for the I/R brain injury beyond the conventional therapeutic time window. We investigated the amount of hEPO delivered into the sonicated brain tissues and the effectiveness in neuroprotection.

Focused ultrasound sonication with microbubbles could effectively boost the vascular permeability and then extend the therapeutic time window of EPO as well as its neuroprotective effects in both acute and chronic phases after I/R injury. In the acute phase, the total sonication volume was smaller than the size of infarction and hence the enhancement of hEPO delivery was only beneficial to part of the infarcted brain. As shown in Fig. 2A, the concentrations of hEPO in sections 3 and 4 (the sonicated region) were significantly higher, and the TTC staining showed that the infarct volume was reduced over 50% as compared with the control or I/R+hEPO groups (Fig. 3B). Furthermore, in the chronic phase, both limb-use asymmetry and dynamic gait test for the evaluation of the chronic behavioral recovery showed that there was a significant improvement for the hEPO+MBs/FUS treatment. The chronic loss of brain cortex was reduced by the hEPO+MBs/FUS treatment (Fig. 5A). These results indicated that MBs/FUS enhanced the hEPO entry even 5 h after I/R, which resulted in neuron protection in both acute and chronic phases. Although stroke itself might alter hEPO delivery, the amount of hEPO entering the infarction area did not produce significant therapeutic effect. As hEPO combined with MBs/FUS, it can result in a significant neuroprotection on both acute and chronic phases.

It has been demonstrated that intracerebraventricular administration of hEPO inhibits the I/R-induced brain injury [30]–[32]. However, direct injection of hEPO into the brain is not a practical approach to have an appropriate hEPO distribution in the entire infarcted region. In the meanwhile, this kind of interstitial method can result in severe hemorrhages and brain trauma. On the contrary, systemic delivery of hEPO can have a much more uniform distribution of hEPO in the infarcted volume but may be limited by the therapeutic time window. In this study, transcranial, noninvasive FUS technology was demonstrated to be a useful modality to transiently open the localized BBB for the targeted delivery of neuroprotectant to treat the ischemic stroke-induced brain injury beyond the conventional therapeutic time window.

Brines et al. reported that animals receiving hEPO ∼3 h after occlusion (equal to 2 h after reperfusion) showed significant reduction of necrosis volume compared with controls [33]. Animals receiving hEPO 6 h after occlusion (equal to 5 h after reperfusion) exhibited a significant decrease in injury volume, but the effect was substantially smaller compared with animals receiving hEPO earlier. Gan et al. reported that EPO exerted significantly neuroprotective effects when administered up to 4 h after I/R in MCAO model, but the effects were significantly diminished and lost when administered 6 h after I/R [34]. In our study, we employed 3VO for 50 min and injected EPO at 5 h after reperfusion and the result showed that there was no significant neuroprotection. These might be due to different stroke models with various occlusion and ischemic duration would produce different levels of impact on the brain.

EPO-TAT administered at the onset of post-stroke reperfusion [35] showed the ability across the BBB for neuroprotection. Derivatives of EPO such as CEPO had the neuroprotection ability only within 4 h after occlusion, which is equal to 3 h after reperfusion, in a rat model of focal ischemia [36]. BBB is more permeable for these EPO derivatives within 3 h after reperfusion due to leaky vascularity. Mutant EPO exerted neuroprotective effects up to 4 h after reperfusion but gradually lose its efficacy as time went by [34]. However, the neuroprotective effects were diminished and lost when the mutant EPO was administered 6 h after reperfusion. Ischemia is an acute pathological process and cells die rapidly within first several hours after ischemia. Therefore, neuroprotective drugs must be delivered within their therapeutic window. In this study, we demonstrated that MBs/FUS had the ability to enhance EPO into the brain at 5 h after reperfusion (∼6 h after the onset of occlusion). MBs/FUS can open the intact BBB and extend the therapeutic time window of EPO.

The parameters used in this study are based on our previous work [14], which is able to minimize the brain tissue damage. Ultrasound pressure would have caused the microbubbles in the acoustic beam oscillation and even cavitation during sonication [37], [38]. These oscillation and cavitation may open vascular walls to enhance hEPO transport into brain tissues. However, the above phenomena may produce some small hemorrhages for the brain tissue in the focal zone, which might cause some damage. To achieve effective drug delivery and minimize this side-effect, we can control acoustic pressure, duty cycle, sonication time, MB dose, etc.

For clinical patient treatments, FUS transducers should be combined with magnetic resonance imaging (MRI) system and thus the MR imaging can be used to guide the FUS transducer to have a precision sonication and to monitor the treatment response. Recently, it has been shown the feasibility of using MRI-guided FUS with MBs to noninvasively open the localized BBB on non-human primates [39], [40]. In this preliminary study, the neuroprotective agent was used only one dose and one time. It requires further scrutiny and examination for the combination of FUS sonication with multiple treatments of neuroprotectants.

MBs/FUS can transcranially and transiently open the localized BBB for the transport of macromolecular drug into the desired brain region. In this study, we utilized this modality for the localized delivery of neuroprotective agent into the infarcted brain of rats beyond the conventional therapeutic time window. The results of acute and chronic investigation show that this modality can provide an alternative treatment option to deliver neuroprotectants or drugs to the injured brain.
